# Mercury in fur of Daubenton’s bat (*Myotis daubentonii*) in Southern Sweden and Comparison to Ecotoxicological Thresholds

**DOI:** 10.1007/s00128-017-2206-3

**Published:** 2017-11-11

**Authors:** Staffan Åkerblom, Johnny de Jong

**Affiliations:** 1 Department of Aquatic Sciences and Assessment, Swedish University of Agricultural Sciences, 750 07 Uppsala, Sweden; 2 Swedish Biodiversity Centre, Swedish University of Agricultural Sciences, Box 7016, 750 07 Uppsala, Sweden; 3 Department of Aquatic Sciences and Assessment, Swedish University of Agricultural Sciences, Lennart Hjelms väg 9, 756 51 Uppsala, Sweden

**Keywords:** Daubenton’s bat, Mercury, Ecotoxicological thresholds, Sweden

## Abstract

To characterise mercury (Hg) exposure in Daubenton’s bat (*Myotis daubentonii*, Kuhl 1817) in southern Sweden, 17 specimens were captured in 2013 and back fur samples were taken for analysis to determine Hg concentrations. The fur Hg levels determined [1.15 ± 0.27 (mean ± standard deviation, n = 17) µg Hg g^−1^ fresh weight (fw)] represent a baseline for comparison in future assessments of Hg exposure in bat populations in northern Europe. Mercury concentrations were close to those reported in fur from other bat species, but were lower than proposed toxicological thresholds in bats (> 30 µg Hg g^−1^ fw) and mice (5 µg Hg g^−1^ fw). This is the first study to examine Hg exposure in bats in Scandinavia.

Mercury (Hg) has severe impacts as an environmental pollutant, with aquatic ecosystems in particular functioning as an important route for Hg exposure in wildlife (Chan et al. 2003; Driscoll et al. 2013). The situation is especially severe in Sweden, since the environmental quality standard (EQS) of 0.02 mg kg^−1^ wet weight set to protect ecosystem health under the EU Water Framework Directive is exceeded 10- to 100-fold in aquatic biota across all water bodies (Åkerblom et al. 2014). The EQS is mainly intended to protect wildlife feeding on aquatic biota from secondary poisoning, so it is also important to determine the extent to which wildlife is exposed. Bats foraging in aquatic habitats bioaccumulate Hg by eating insects that emerge from the water (Wolfe et al. 1998; Wada et al. 2010; Little et al. 2015a). Compared with terrestrial foraging bats, aquatic foraging bats have adapted by developing higher levels of protective proteins (metallothioneins), which are used to prevent ecotoxicological effects from heavy metal contamination (Pikula et al. 2010). Owing to the high Hg levels in Swedish aquatic ecosystems, there may be severe Hg exposure in bat populations that forage in regions of Sweden with high Hg levels in aquatic biota. Despite the potential for Hg exposure in bats, this issue has not previously been studied in Sweden. This study rectifies this knowledge gap and provide a baseline that can be used for comparison in future studies. Elsewhere, research focusing on Hg exposure of bats is increasing (Hickey et al. 2001; Zukal et al. 2015).

Bats can be used as bio-indicators, and for this reason bat population mapping is an important component of environmental monitoring programmes. One of the aims of monitoring is to detect heavy metal exposure in bat populations and mitigate remediation efforts (Zukal et al. 2015). Collection of fur to analyse contamination level is a good non-lethal method for determination of heavy metal exposure in bats (Flache et al. 2015; Hernout et al. 2016b). The use of fur from the back of the bat provides heavy metal data that have been shown to be correlated with heavy metal data for other organs, e.g., blood and liver (Massa and Grippo 2000; Nam et al. 2012; Zukal et al. 2015; Hernout et al. 2016b). The collection and analysis of fur also provides the potential to estimate possible ecotoxicological effects by comparison against toxic thresholds.

Environmentally relevant Hg exposure impairs the function of the nervous system and behaviour in mammals (Clarkson and Magos 2006). Toxic effects in bats from Hg exposure include impairment of neurological function [toxic threshold in fur = 100 µg g^−1^ fresh weight (fw)] (Nam et al. 2012), while damage to mitochondrial DNA has also been reported (toxic threshold in fur = 30 µg Hg g^−1^ fw) (Karouna-Renier et al. 2014). Shifts in ambulatory activity in wild mouse populations and toxicological effects have been found at fur levels > 5 µg Hg g^−1^ fw (Burton et al. 1977). There is also a risk of reproductive disorders, with reports of oxidative stress in testes of e.g., rats following Hg exposure (Boujbiha et al. 2009; Burton and Meikle 1980). The effects of Hg also include developmental alterations in the foetus that can cause impairment or even death after birth (Scheuhammer et al. 2007).

Daubenton’s bat (*Myotis daubentonii*, Kuhl 1817) is common in Sweden, with a distribution that extends above the Arctic circle, and is therefore useful for comparison of Hg exposure between regions (Ahlén 2011; Siivonen and Wermundsen 2008). The aim of this study was to determine the concentrations of Hg in fur from Daubenton’s bat, of both sexes and different stages of maturation, at two sites in southern Sweden that are not subject to local sources of Hg pollution or other disturbances. The hypothesis tested was that Hg concentrations in the fur of Daubenton’s bat are below the reported EQS for Hg in bats. We also tested whether variation in Hg concentration in the fur of Daubenton’s bat could be explained by sex and maturation stage (adult/juvenile). The study was intended to provide a general indication of Hg exposure and to estimate the threat posed by Hg exposure in Swedish populations of Daubenton’s bat.

## Materials and methods

Bats were trapped in a mist net placed above the water surface in small streams on 22 July and 23 July 2013 at two different sites: Stockamöllan (coordinates: 55°56′N, 13°22′E) and Södra Åsum (coordinates: 55°38′N, 13°42′E). Both sites are located in the province of Skåne, southern Sweden. Fur samples were collected in conjunction with annual surveillance for the prevalence of bat lyssavirus type 2. Bats were classified as either juvenile or adult based on examination of finger bones (Anthony 1988). Fur samples were clipped from the back of the bats using stainless steel surgical scissors. The samples were stored in plastic tubes and kept frozen (− 18°C) during transport to the laboratory and until analysis. Immediately after fur sampling, the bats were released.

In total, 17 individuals were captured and used for fur sampling. These comprised 7 males and 10 females (Table 1). Thirteen of these individuals were captured at the Södra Åsum site, while only four individuals were captured at Stockamöllan. Bat fur specimens from both adults and juveniles were collected at Södra Åsum, but only adult bats were sampled at Stockamöllan.

**Table 1:** Concentration of mercury (Hg) in fur taken from Daubenton’s bat specimens captured at two sites in southern Sweden

Fur Hg concentration in Daubenton’s bat specimens					
Site	Sex	Age^a^	Min/max (µg g^−1^ fw)	Mean ± SD^b^ (µg g^−1^ fw)	N
Stockamöllan	Male	Juvenile	0.44/0.49	0.46 ± 0.04	2
		Adult	1.49/1.66	1.57 ± 0.12	2
	Female	Juvenile	0.51/1.30	0.82 ± 0.56	2
		Adult	0.47/1.44	0.84 ± 0.33	7
Södra Åsum	Male	Juvenile	–	–	–
		Adult	1.23/2.10	1.65 ± 0.44	3
	Female	Juvenile	–	–	–
		Adult	2.31/2.31	2.31	1
Total			0.44/2.30	1.15 ± 0.27	17

^a^Bats were classified as either juvenile or adult based on examination of finger bones (Anthony 1988)

^b^Weighted arithmetic mean ± standard deviation

Fur was analysed for Hg concentration using an SMS-100 Mercury Analyzer (Perkin Elmer) by thermal decomposition (750°C) followed by amalgamation on a gold trap, thermal desorption and analysis of vapour Hg by atomic absorption spectroscopy (AAS) according to EPA method 7473. Fresh weight of the bat fur samples, determined using a balance with a detection limit of 0.05 mg and weight varied between 2 and 30 mg. The total Hg content in the samples ranged between 0.71 and 3.87 ng Hg (the reported detection limit for the method is 0.005 ng Hg). Blank samples (empty sample boats) were analysed regularly (mean Hg content < 0.01 ng Hg). The accuracy of the Hg analysis was tested at regular intervals during the analysis using fish liver (CNRC DOLT4) and lake sediment (IAEA SL-1) reference material (RM) with a certified concentration of 2.58 µg Hg g^−1^ dry weight and 0.13 µg Hg g^−1^ dry weight, respectively. Recovery of RM was 105% ± 2% [mean ± standard deviation (SD)]. The amount of sample material collected could only be used for one analysis of each bat specimen. Analysis of hair replicate samples typically has a precision higher than 97% relative SD (Gashaw et al. 2017).

A weighted arithmetic mean for the Hg concentration of fur samples were calculated based on data from each category of bat (adult/juvenile, female/male, Södra Åsum/Stockamöllan). Differences in Hg concentration in fur of Daubenton’s bat were tested by the non-parametric median test using a 1-way test using all groups available (males/females, adult/juvenile, and Stockamöllan/Södra Åsum). All tests were evaluated for significance at α = 0.05. Statistical calculations were executed in JMP 11.0 (SAS Institute Inc., Cary, NC, USA).

## Results and Discussion

**Fig. 1:**
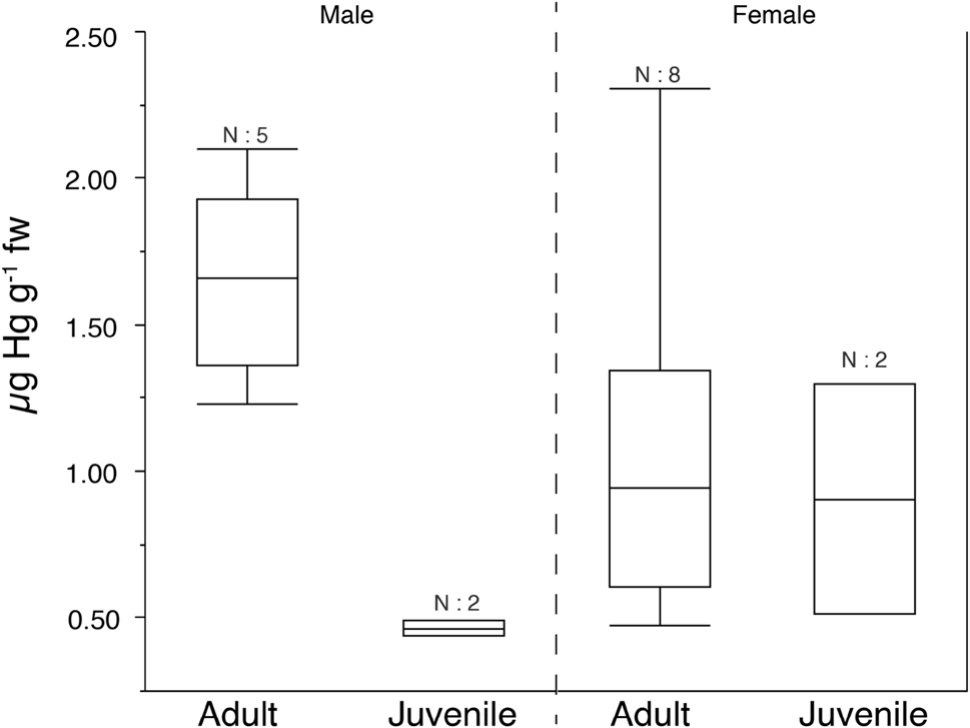
Box and whiskers plot of concentrations of Hg in back fur (µg Hg g^−1^ fw) from adult and juvenile male and female specimens of Daubenton’s bat (*Myotis daubentonii*) captured at two sites in southern Sweden (Stockamöllan and Södra Åsum). Box plots (Bars = 10 and 90 percentiles; boxes = 25 and 75 percentile; vertical line = median). *N* number of specimens

The Hg concentration in fur from the Daubenton’s bat specimens varied between 0.44 and 2.30 µg Hg g^−1^ fw, with an overall weighted arithmetic mean of 1.15 µg Hg g^−1^ fw (SD = 0.27 µg Hg g^−1^ fw) with no significant difference between the sampled groups (median test: χ^2^ = 10.9, df = 5, *p* = 0.054) (Table 1; Fig. 1). Fur samples were not washed prior the Hg analysis to remove any external contamination (Flache et al. 2015). By not removing possible exogenous contaminants by washing of the fur samples possible artefacts in the data set can have been introduced, e.g. the high Hg concentration in adult female bat specimen at Södra Åsum (2.31 µg Hg g^−1^ fw). Data on fur Hg concentration in the present study represented only two populations of bats with a small number of observations (n = 17) and skewness in the number of observations between the studied groups (Table 1). The data still provides an indication of fur Hg concentrations that can be expected in the study region.

Compared with fur Hg concentration reported for other regions and continents (Table 2), bat populations in southern Sweden were exposed to Hg to approximately the same extent as in regions in North America and Asia (Table 2). There are differences between bat species in the published data, with big brown bat (*Eptesicus fuscus*) having the lowest levels of Hg, while little brown bat (*Myotis lucifugus*) has the highest reported levels (although those high values were detected at polluted sites) (Nam et al. 2012).

**Table 2:** Reported fur mercury (Hg) concentrations in bats of different species, ages and sexes at reference and polluted sites in Europe, Asia, and North America (data in this study shown for comparison)

Fur Hg concentration in bat species						
Species	Sex	Age	Site	Min–max (µg Hg g^−1^ fw^a^)	Mean ± SD/SE (µg Hg g^−1^ fw^a^)	References
*Myotis daubentonii*	M/F	J/A	Southern Sweden	0.4–2.3	1.2 ± 0.3	This study
*Rhinolophus cornutus cornutus*	n.i	n.i	Japan, Subashiri		2.6 ± 0.4^b^	Miura et al. (1978)
	n.i	n.i	Japan, Mine		3.5 ± 1.3	
	n.i	n.i	Japan, Owase		5.0 ± 2.2	
	n.i	n.i	Japan Bato		6.3 ± 4.3	
*Rhinolophus ferrum-equinum*	n.i	n.i	Japan, Hiramatsu		7.6 ± 3.4	
	n.i	n.i	Japan, Mine		5.1 ± 0.8	
	n.i	n.i	Japan, Nagashima		5.1 ± 1.7	
	n.i	n.i	Japan, Ise		6.8 ± 2.0	
*Miniopterus schreibersi fuliginosus*	n.i	n.i	Japan, Mine		10.5 ± 0.9	
	n.i	n.i	Japan, Numazy		10.2 ± 1.7	
*Pipistrellus abramus*	n.i	n.i	Japan		33.0 ± 6.3	
*Vespertilio superans*	n.i	n.i	Japan		33.7 ± 4.2	
*Myotis lucifugus*	F	A	Virginia USA, Reference site	1.4–5.5	3.1 ± 1.3	Nam et al. (2012)^c^
	F	A	Polluted site	7.3–274	132 ± 94	
*Eptesicus fuscus*	F	A	Virginia USA, Reference site		10.9 ± 0.5	Wada et al. (2010)^c^
	F	A	Polluted site	4.8–65.4	28.0 ± 4.1	
*Myotis lucifugus*	F	A	Canada, Nova Scotia	0.8–47.0	9.3	Little et al. (2015a, b)^a^
	F	A	Canada, Prince Edward Island	1.6–15.2		
	F	A	Canada, Newfoundland	1.0–28.3		
	F	A	Canada, Labrador	0.6–23.4		
*Myotis lucifugus*	n.i	n.i	Ontario, Canada		1.5	Hickey et al. (2001)
*Eptesicus fuscus*	n.i	n.i			1.5	
*Myotis leibii*	n.i	n.i			5.3	
*Myotis septentrionalis*	n.i	n.i			4.4	
*Perimyotis subflavus*	n.i	n.i	Northeastern USA	Max = 2.8	0.7	Yates et al. (2013)
*Myotis lucifugus*	n.i	n.i		Max = 3.8	0.3	
*Myotis septentrionalis*	n.i	n.i		Max = 3.7	0.6	
*Lasiurus borealis*	n.i	n.i		Max = 0.9	0.1	
*Lasiurus cinereus*	n.i	n.i		Max = 0.03	0.02	
*Miniopterus schreibersii*	n.i	A	Murcia province, Spain	0.41–2.27	1.13 ± 0.48	Lison et al. (2017)
	M	n.i		0.68–1.95	1.20 ± 0.39	
	F	n.i		0.41–2.27	1.05 ± 0.57	
	F	A		0.41–2.27	0.96 ± 0.63	
	F	A		0.50–1.68	1.19 ± 0.50	

^a^Fur Hg levels are reported as range (min–max) or mean with standard deviation (SD) or standard error (SE)

^b^Specimen preserved in alcohol in the National Science Museum, Tokyo

^c^Polluted sites: Hg contamination downstream from a former textile factory at Renkin Barn (South River) with estimated daily release of up to 100 pounds (45.3 kg) of mercuric sulphide from the factory

*M* male, *F* female, *J* juvenile, *A* adult, *n.i*. no information

Sex and age represent important sources of variation in Hg bioaccumulation in wild populations of bats (Yates et al. 2013). In Sweden, we have observed sex-biased differentiation in Daubenton’s bat, as small streams and ponds are more commonly used by males while the females use lakes and rivers for foraging, although small streams close to lakes may be used by both sexes (unpublished data). Lower fur Hg concentrations in females bats compared with male bats have been explained by the depuration of Hg during lactation (Lison et al. 2017; Yates et al. 2013) even though it was not possible to detect any difference between sexes in this study. Differentiation between sexes in their foraging and roosting strategies could also add to differences in Hg concentrations between bat populations. Juvenile bats of both sexes forage close to the colony in the same area as the female, especially during lactation, while there are clear differences in foraging strategies between male and female adult Daubenton’s bat (Dietz et al. 2006). Males and females also differ in their strategy of selecting roosting places, with landscape factors (altitude, abundance of ponds, lakes and rivers) playing an important role (Encarnação et al. 2005). New growth fur should be targeted for sampling and analysis of endogenous markers in bats. Moulting cycles in bats are both age and sex-specific and add to the observed variation in endogenous markers (Fraser et al. 2013). Sampling of fur in this study was done in early-mid summer and was earlier then the timing of new-fur growth that generally occur in late summer-fall. Monitoring protocols should acknowledge the above-mentioned factors for sampling of bat populations in the future.

The Hg levels detected in Daubenton’s bat in southern Sweden were 5- to 100-fold lower than the suggested threshold at which genotoxic (30 µg Hg g^−1^ fw) (Karouna-Renier et al. 2014) or neurological (100 µg Hg g^−1^ fw) (Nam et al. 2012) effects may occur. However, a much lower threshold (5 µg Hg g^−1^ fw) has been proposed for behavioural effects in wild mouse populations (Burton et al. 1977). Those toxicity studies were performed at contaminated sites and the Hg exposure in bats at Hg-contaminated sites in Sweden may well reach toxic levels. Bats caught near rivers and lakes with fish that exceed consumption advisory levels (1 mg kg^−1^ wet weight) in Arkansas (USA) had fur Hg concentration ranging from 1 to 30 µg Hg g^−1^ fw (Massa and Grippo 2000). Fish Hg levels above these levels have been found in a large proportion of lakes in Sweden (Åkerblom et al. 2014). Despite the uncertainty resulting from the small number of observations, we speculate that a linear relationship between aquatic biota and Hg exposure in Daubenton’s bat across ecosystems could eventually lead to Hg concentrations that are 2- to 20-fold higher in northern Sweden than at southern Swedish sites.

This study on Hg exposure in bats is the first in Scandinavia and provides an estimate for future comparisons on Hg exposure in bat populations in other regions in northern Europe. It also provides an estimate of the environmental stress on bat populations and the potential risk of Nordic bats being affected by the ecotoxicological effects of Hg (Zukal et al. 2015). This type of data is important, since heavy metal exposure is considered to play an important role for the health of bat populations and is thus relevant in monitoring work on bats (Hernout et al. 2016a; Luftl et al. 2003; Walker et al. 2007; Zukal et al. 2015).

The extent to which Hg exposure affects the health of bat populations can only be estimated by comparison of field-collected data with toxic thresholds. The results presented here are important for future establishment of a broader environmental monitoring programme on Hg exposure in bat populations in Sweden. The data also provide a reference for future studies on Hg exposure, risk assessments and decision making to protect the health of bat populations from negative effects of mercury.
